# Pan-cancer analysis and experimental validation revealed the m6A methyltransferase KIAA1429 as a potential biomarker for diagnosis, prognosis, and immunotherapy

**DOI:** 10.18632/aging.204968

**Published:** 2023-08-21

**Authors:** Chao Ma, Qiming Zheng, Yepeng Wang, Guoxiang Li, Mengmeng Zhao, Zhigang Sun

**Affiliations:** 1 Department of Thoracic Surgery, Central Hospital Affiliated to Shandong First Medical University, Jinan 250013, Shandong, China; 2 School of Clinical Medicine, Weifang Medical University, Weifang 261053, Shandong, China; 3 Jinan Central Hospital, Shandong University, Jinan 250013, Shandong, China; 4 Research Center of Translational Medicine, Central Hospital Affiliated to Shandong First Medical University, Jinan 250013, Shandong, China

**Keywords:** KIAA1429, pan-cancer, diagnosis, prognosis, immune infiltration

## Abstract

Background: KIAA1429, also known as VIRMA (vir-like m6A methyltransferase associated), plays a crucial role in tumorigenesis by modulating the level of m6A methylation. Previous studies have reported the prevalent overexpression of KIAA1429 in multiple cancers, related to a poor prognosis. Nevertheless, the precise role of KIAA1429 in tumor progression and its impact on the immune response remains unclear.

Methods: A differential analysis of KIAA1429 expression was performed across cancers using data from the Cancer Genome Atlas (TCGA) and Genotype-Tissue Expression (GTEx) databases. We evaluated the role of KIAA1429 in the diagnosis, prognosis, and immunotherapy of tumor patients using bioinformatics methods. In addition, we also analyzed the associations between KIAA1429 and DNA methylation, immunotherapy. RT-qPCR was used to study the expression levels of KIAA1429 mRNA in 11 cell lines.

Results: KIAA1429 is found to be overexpressed in 28 cancer types, but its expression is relatively low in patients with acute myeloid leukemia (LAML) and ovarian serous cystadenocarcinoma (OV). Moreover, KIAA1429 demonstrates a positive correlation with advanced stages of multiple cancers. Kaplan-Meier (KM) analysis suggested that patients with elevated KIAA1429 expression had shorter survival. Furthermore, KIAA1429 shows strong associations with DNA methylation, tumor-infiltrating immune cells (TIICs), and the tumor microenvironment (TME). RT-qPCR results indicated significantly higher expression of KIAA1429 in tumor cells compared to matched-normal cells.

Conclusions: In summary, our work illustrates that KIAA1429 expression is positively connected with poor prognosis in multiple cancers. Moreover, KIAA1429 could serve as a diagnostic factor and a predictor of immune response for specific tumor types.

## INTRODUCTION

Tumors pose a serious threat to human life and health [1, 2]. Currently, cancer treatment is evolving toward personalization and precision [3, 4]. However, the overall survival (OS) rate of tumor patients has not improved significantly. Notably, immunotherapy has become a very important tumor treatment that can significantly prolong the survival time of patients with lung adenocarcinoma (LUAD) [5], and skin cutaneous melanoma (SKCM) [6]. In addition, with the development of bioinformatics, pan-cancer analysis based on TCGA and GEO databases can help discover potential diagnostic, prognostic, and immunotherapeutic biomarkers by analyzing the biological significance of target genes in multiple tumors [7, 8].

m6A modifications, primarily distributed in mRNA and non-coding RNA, are among the most abundant chemical modifications in eukaryotes [9–11]. Functionally, m6A methylation plays a crucial role in various biological processes, such as obesity, infertility, and tumorigenesis, by modulating RNA stability, splicing, export, translation, and decay [12]. This dynamic and reversible process is mediated by methyltransferases for addition, demethylases for removal, and RNA-binding proteins for recognition [13].

There is mounting evidence suggesting the involvement of KIAA1429, a key component of the methyltransferase complex, in tumorigenesis, and it is significantly associated with a poor prognosis [14, 15]. Zhou et al. discovered that KIAA1429 enhances cell growth and migration in colon adenocarcinoma (COAD) by upregulating SIRT1 expression through the stabilization of its mRNA [16]. KIAA1429 acts as an oncogene in stomach adenocarcinoma (STAD). Mechanistic analysis revealed that KIAA1429 promotes cell proliferation by inducing an increase in c-Jun expression in an m6A manner [17]. The study by Zhang et al. suggested that KIAA1429 overexpression, driven by gene amplification, contributes to the progression of LUAD by downregulating BTG2 expression through the reduction of its mRNA stability in an m6A manner. These findings demonstrate the oncogenic role of KIAA1429 in different cancers and highlight the importance of m6A methylation in tumorigenesis [18].

Previous research has predominantly focused on investigating the role of KIAA1429 in a single type of tumor. However, no comprehensive analysis of KIAA1429 in pan-cancer has been conducted. Therefore, we examined the association between KIAA1429 expression and prognosis, DNA methylation, and immune cells. Subsequently, we conducted co-expression analyses between immune-related genes and KIAA1429, along with an enrichment analysis, to explore the biological significance of KIAA1429 across cancers. Our findings indicate that KIAA1429 shows promise as a diagnostic and prognostic biomarker as well as a predictor of immune response.

## MATERIALS AND METHODS

### Data processing and differential expression analysis

RNA-seq and clinical data were obtained from the TCGA and GTEx databases, while data from each tumor cell line was retrieved from the CCLE database. All RNA-seq data underwent a log2 transformation. The differential analysis between tumors and matched-normal tissues was performed using R Studio (Version 4.2.1). The results were visualized using the R package “ggplot2”.

### Immunohistochemistry (IHC) staining

The expression levels of the target gene in various human tissues and organs were investigated using the HPA database, which integrates proteomics and transcriptomics data [19]. Based on the HPA database, we acquired IHC images of the KIAA1429 protein in four types of tumors and their corresponding normal tissues, including COAD, diffuse large B-cell lymphoma (DLBCL), head and neck squamous cell carcinoma (HNSC), and testicular germ cell tumors (TGCT).

### Relationship between KIAA1429 expression and prognosis, TNM stage, and pathological stage

Clinical data on tumor patients was extracted from the TCGA database. The Cox regression model was constructed by the R packages “survival” and “forestplot.” The correlation of KIAA1429 expression with OS, disease-specific survival (DSS), progress-free interval (PFI), and disease-free interval (DFI), was explored using the R packages “survival” and “survminer.” An analysis of the correlation between KIAA1429 expression and TNM stage, pathological stage was conducted.

### Correlation between KIAA1429 expression and tumor mutational burden (TMB), microsatellite instability (MSI)

Previous studies have reported that TMB and MSI have the potential to serve as prognostic biomarkers for tumor patients, influencing their response to immunotherapy [20, 21]. In this study, the TMB and MSI scores for each sample were calculated based on somatic mutation data acquired from the TCGA database. The Spearman correlation coefficient was employed to investigate the correlation between the expression of KIAA1429 and TMB as well as MSI. The results were visualized utilizing the R packages “ggradar” and “ggplot2”.

### ROC curve for KIAA1429 expression in different cancers

The receiver operating characteristic curve (ROC) is a graphical tool utilized for assessing the performance of a classifier. It demonstrates the trade-off between sensitivity (true positive rate) and specificity (true negative rate) of the classifier at various thresholds. The area under the curve (AUC) obtained from the ROC analysis is used to evaluate the diagnostic accuracy of KIAA1429 in various types of cancer.

### Correlation between KIAA1429 expression and tumor immunity

In this study, we conducted an ESTIMATE analysis based on the transcriptional profiles of tumor samples to calculate the abundance of tumor cells, stromal cells, and immune cells using the R package “estimate” [22]. Moreover, we examined the association between KIAA1429 expression and StromalScore, ImmuneScore, and ESTIMATEScore.

The CIBERSORT database is the most widely used tool for analyzing TIICs because it can assess the proportions and abundances of 22 immune cell types in the TME. We employed the R package IOBR to evaluate the infiltration scores of the 22 TIICs in different tumors [23]. The association between KIAA1429 expression and TIICs was investigated through Pearson's correlation analysis. The results were visualized using the R packages “gcookbook” and “ggplot2”. Furthermore, the correlation between KIAA1429 expression and immune-related genes was examined using the Spearman method.

### Correlation between KIAA1429 expression and DNA methylation

DNA methylation plays a crucial role in tumorigenesis and progression by influencing the expression of oncogenes and activating key oncological pathways [24, 25]. Data on DNA methylation was retrieved from the TCGA database. An analysis was conducted to examine the relationship between KIAA1429 expression and DNA methylation. The association between KIAA1429 and clinical outcomes was investigated through KM analysis.

### GESA and GSVA

To explore the underlying biological processes associated with KIAA1429 expression across cancers, we conducted GSEA and GSVA utilizing gene sets representing functional categories and pathways obtained from the official GSEA website. For the GSEA analysis, we employed the following R packages: “ReactomePA,” “org.Hs.eg.db,” “clusterProfiler,” and “enrichplot”.

GSVA, which was conducted by the R package “GSEABase,” “limma,” “GSVA,” “ggplot2,” “ggthemes,” and “ggprism,” was used to examine the differences in pathway activity scores between groups with high and low KIAA1429 expression.

### Immunotherapy prediction and drug sensitivity analysis

Mounting evidence suggests that immunotherapy, represented by immune checkpoint inhibitors (ICIs), is a prominent cancer treatment, significantly improving patient prognosis [26–28]. We chose the IMvigor210 cohort (bladder urothelial carcinoma, BLCA) to validate the role of KIAA1429 expression in immunotherapy. The relationship between KIAA1429 expression and OS was investigated using KM analysis. The predictive performance of KIAA1429 expression was assessed using the Chi-square test. Finally, we performed a drug sensitivity analysis to investigate the association between KIAA1429 expression and the IC50 value of anti-cancer drugs based on the CellMiner database.

### Cell culture

BEAS-2B (human bronchial epithelial cells), SV-HUC-1 (human uroepithelial cells), HK-2 (human renal tubular epithelial cells), A549 and H1299 (LUAD cells), T24, MGH-U3, and BIU-87 (BLCA cells), 786-O, Caki-1, and 769-P (kidney renal clear cell carcinoma, KIRC cells) were derived from the Cell Bank of the Chinese Academy of Sciences. The BEAS-2B, H1299, BIU-87, 786-O, and 769-P cell lines were cultured in 1640 medium. The A549 cell line was cultured in DMEM medium. The MGH-U3 cell line was cultured in DMEM/F12 medium. The T24 and Caki-1 cell lines were cultured in M5A medium. The SV-HUC-1 cell line was cultured in F12K medium, and the HK-2 cell line was cultured in F12 medium. All the media were supplemented with 10% FBS and penicillin/streptomycin.

### Real-time quantitative polymerase chain reaction (RT-qPCR)

To examine KIAA1429 mRNA expression levels in various cell lines, total RNA was extracted from cell lines using Trizol Reagent (Thermo Fisher Scientific) and reverse transcribed into cDNA according to the manufacturer’s protocol. The SYBR Green Master Kit (Vazyme) was then used to perform RT-qPCR on a LightCycler 480 II (Roche Diagnostics) instrument. The sequences of primers were as follows: KIAA1429: F: CGAGCGCTGAGCAAAGTTC, R: CAGCCTCTTAGCACCAGACC. β-actin: F: CCCATCTATGAGGGTTACGC, R: TTTAATGTCACGCACGATTTC.

### Statistical analysis

All RNA-seq data were log2 transformed. The differential analysis of KIAA1429 expression in pan-cancer was conducted using the Wilcox test. Statistical significance was defined as a p-value < 0.05. The correlation between KIAA1429 expression and TME, as well as tumor immunity was analyzed using the Spearman and Pearson correlation methods. All statistical analyses were conducted based on R Studio (Version 4.2.1).

## RESULTS

### Differential analysis of KIAA1429 expression between tumor and normal tissues

**Figure 1:**
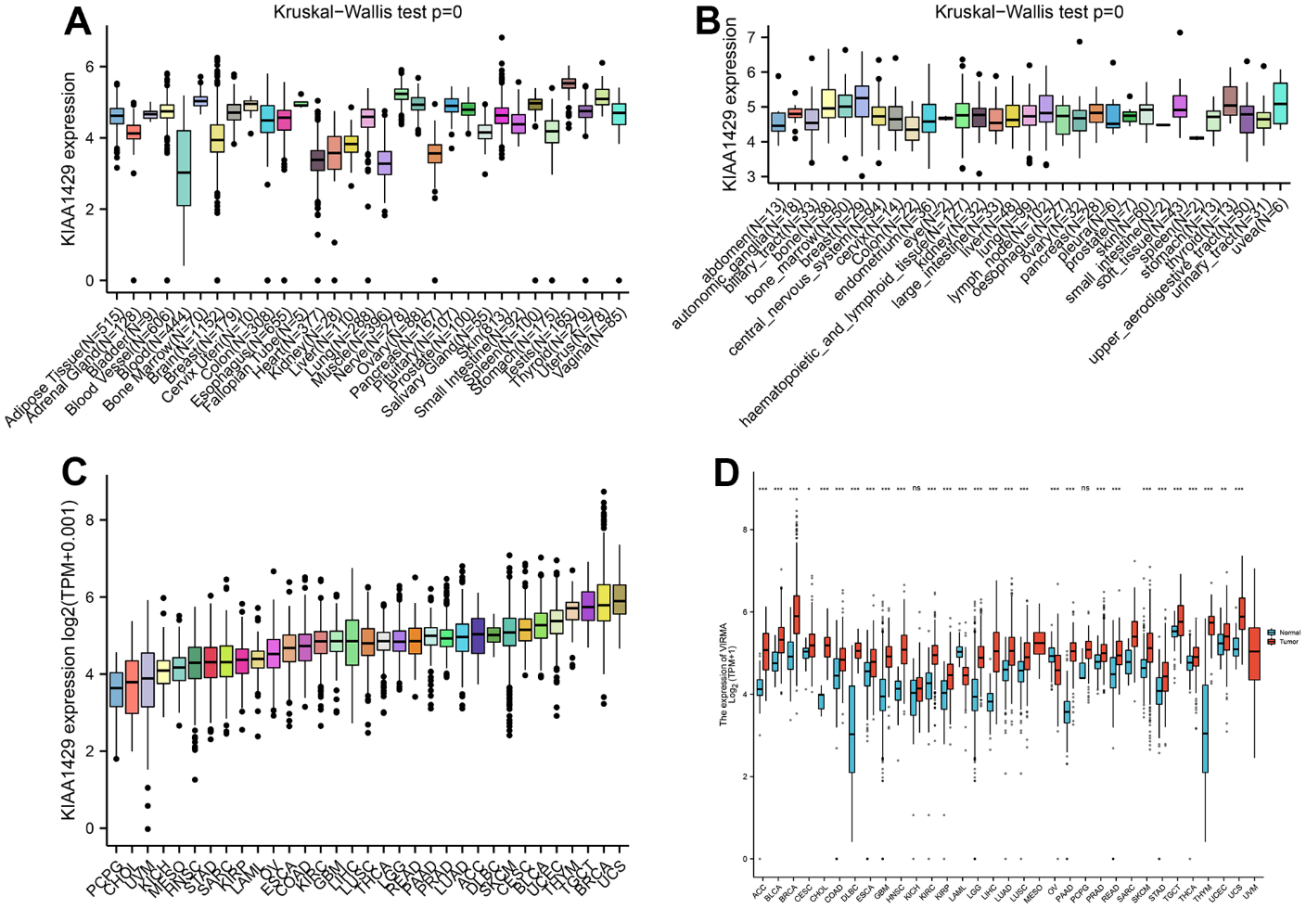
**Differential analysis of KIAA1429 expression.** (**A**) KIAA1429 expression in normal tissues. (**B**) KIAA1429 expression in tumor cell lines. (**C**) KIAA1429 expression in 33 cancer types. (**D**) Comparison of KIAA1429 expression between tumor and normal samples. *P < 0.05, **P < 0.01, ***P < 0.001.

**Figure 2:**
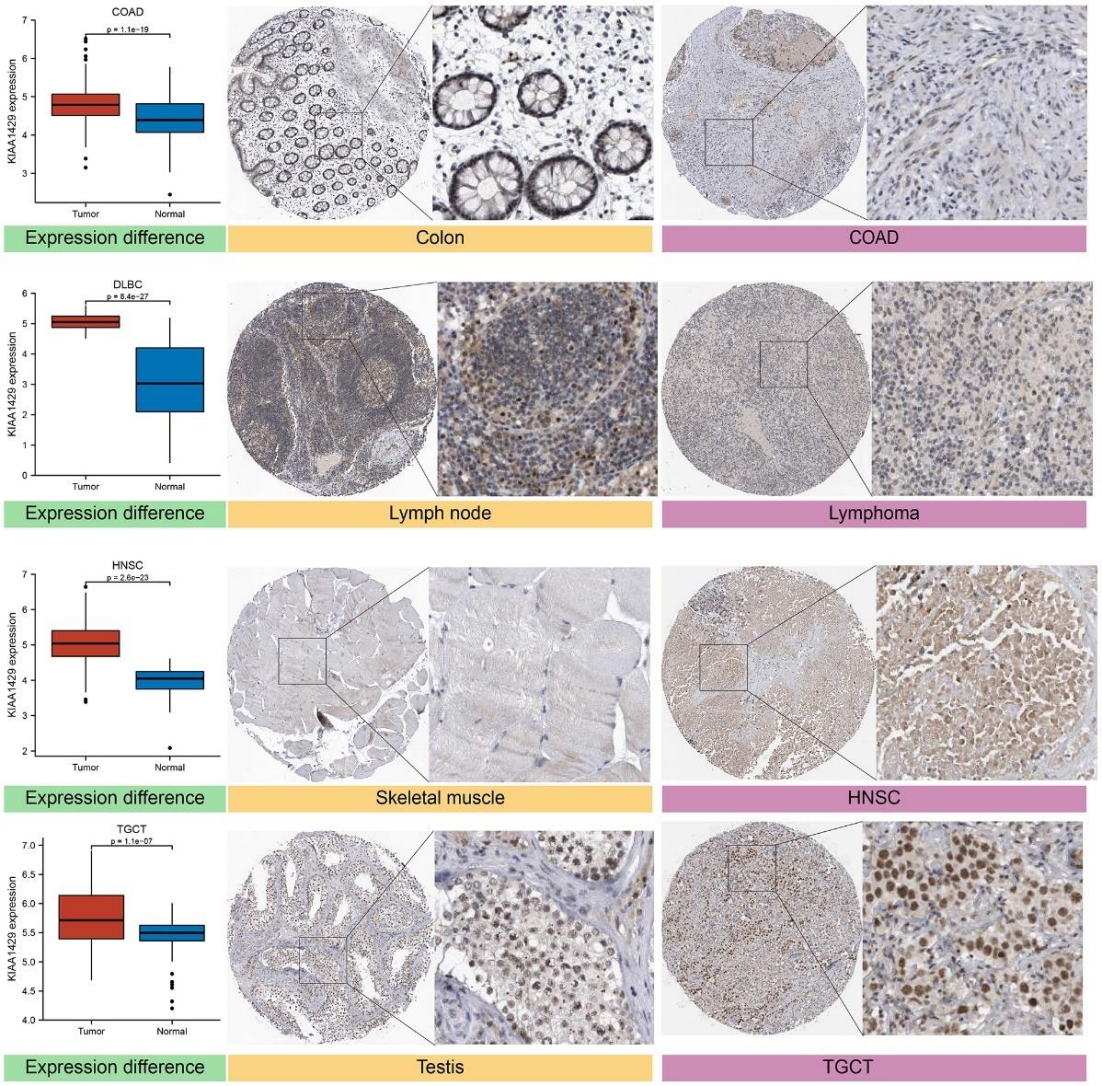
Differential expression of KIAA1429 at mRNA and protein levels in normal and tumor tissues.

Based on the GTEx database, we examined the expression levels of KIAA1429 in 31 normal tissues (Figure 1A). The results indicate that KIAA1429 expression is relatively low in the heart, muscles, and blood, while it is relatively high in the testis, nerves, and uterus. Based on the CCLE data, Figure 1B illustrates the relative levels of KIAA1429 expression in various tumor cell lines. Subsequently, we analyzed the relative level of KIAA1429 mRNA in 33 tumor tissues and ranked them from low to high (Figure 1C). Our findings revealed that the highest expression level of KIAA1429 was observed in uterine carcinosarcoma (UCS), whereas the lowest was found in pheochromocytoma and paraganglioma (PCPG). Based on TCGA and GTEx data, we comprehensively studied the differential expression of KIAA1429 between 33 tumor and normal samples. As depicted in Figure 1D, KIAA1429 is upregulated in 28 tumors and downregulated in 2 tumors. However, no significant difference was observed in KIAA1429 expression levels between mesothelioma (MESO), SARC, uveal melanoma (UVM) tissues, and their respective matched-normal tissues due to the lack of available matched-normal tissues. Furthermore, the expression levels of KIAA1429 in kidney chromophobe (KICH) and PCPG did not show a significant difference compared to normal samples. The results obtained from the IHC data indicated that COAD, DLBC, TGCT, and HNSC tissues exhibited moderate or strong IHC staining for KIAA1429, whereas the matched-normal tissues had low KIAA1429 IHC staining (Figure 2).

### Correlation between KIAA1429 expression and prognosis

We established a Cox regression model to examine the association between KIAA1429 expression and prognosis. A KM survival analysis was performed for each type of cancer, and the statistical analysis utilized the log-rank test. Supplementary Figure 1A demonstrates a significant association between KIAA1429 expression and OS in seven tumors, including UVM (HR=2.23, p=1.6e-3), KIRC (HR=1.72, p=2.7e-3), kidney renal papillary cell carcinoma (KIRP, HR=2.65, p=3.0e-3), hepatocellular carcinoma (LIHC, HR=1.46, p=3.6e-3), adrenocortical carcinoma (ACC, HR=2.91, p=4.4e-3), KICH (HR=10.11, p=4.7e-3), and LUAD (HR=1.46, p=9.3e-3). Further, KM analysis confirms that elevated KIAA1429 expression is associated with shorter OS times in COAD (p=1.8e-3), ACC (p=4.3e-3), KIRP (p=2.1e-4), KICH (p=3.1e-3), KIRC (p=2.5e-3), LIHC (p=2.7e-4), LUAD (p=2.0e-3), MESO (p=3.7e-4), and UVM (p<0.0001) (Supplementary Figure 1B–1J).

The expression of KIAA1429 is correlated with DSS in patients with KIRP (HR=5.82, p=2.5e-5), KIRC (HR=2.35, p=2.5e-4), KICH (HR=14.12, p=4.0e-3), UVM (HR=2.18, p=3.0e-3), MESO (HR=3.09, p=7.7e-3), ACC (HR=2.64, p=0.01), LIHC (HR=1.41, p=0.03), and thyroid carcinoma (THCA, HR=13.32, p=0.02) (Supplementary Figure 1K). The KM analysis reveals that among patients with ACC (p=6.8e-3), COAD (p=7.9e-3), KIRP (p<0.0001), KIRC (p<0.0001), KICH (p=2.3e-4), LIHC (p=0.001), MESO (p=8.6e-4), rectum adenocarcinoma (READ, p=0.028), THCA (p<0.0001), and UVM (p<0.0001) (Supplementary Figure 1L–1U), those with high KIAA1429 expression had a shorter DSS. However, in patients with SKCM (p=0.018), low KIAA1429 expression is positively associated with a longer DSS (Supplementary Figure 1V).

Supplementary Figure 2A illustrates the connection between KIAA1429 expression and DFI in patients with KIRP (HR=7.21, p=9.1e-5), prostate adenocarcinoma (PRAD, HR=3.76, p=3.5e-3), brain lower grade glioma (LGG, HR=2.67, p=4.4e-3), LIHC (HR=1.29, p=0.02), and COAD (HR=3.5, p=0.04). However, in patients with PCPG (HR=0.14, p=0.01), elevated KIAA1429 expression has a positive correlation with a better prognosis. As shown in Supplementary Figure 2B–2F, elevated KIAA1429 expression is predicted to be associated with a shorter DFI in COAD (p=8.4e-3), KIRP (p<0.0001), PRAD (p=1.6e-3), LGG (p=0.013), and LIHC (p=7.2e-3). Conversely, in patients with PCPG (p=9.6e-4), elevated KIAA1429 expression is predicted to be associated with a longer DFI (Supplementary Figure 2G).

Supplementary Figure 2H illustrates that the connection between KIAA1429 expression and PFI in patients with KIRP (HR=5.15, p=4.6e-7), KIRC (HR=2.15, p=5.9e-5), UVM (HR=2.30, p=3.8e-4), PRAD (HR=2.21, p=1.4e-3), LIHC (HR=1.30, p=0.01), KICH (HR=4.16, p=0.01), ACC (HR=1.99, p=0.02), HNSC (HR=1.32, p=0.04), and PCPG (HR=0.34, p=0.04). KM analysis confirms that KIAA1429 elevation is predicted to be a high-risk factor in patients with ACC (p=6.4e-3), DLBC (p=0.02), KIRC (p<0.0001), HNSC (p=5.3e-3), KICH (p=0.014), KIRP (p<0.0001), LIHC (p=7.0e-3), PRAD (p=2.6e-4), UVM (p<0.0001) (Supplementary Figure 2I–2Q). However, KIAA1429 elevation is predicted to be a low-risk factor in patients with PCPG (p=3.6e-3) (Supplementary Figure 2R).

### The diagnosis value of KIAA1429 in different tumors

Our study investigates the relationship between KIAA1429 expression and TNM stage, as well as pathological stage across cancers. The results indicate a significant upregulation of KIAA1429 in the advanced stages of 11 cancer types, namely esophageal carcinoma (ESCA), KICH, KIRP, KIRC, PRAD, MESO, breast invasive carcinoma (BRCA), COAD, HNSC, THCA, and UVM. This observation suggests that increased KIAA1429 expression may serve as a potential biomarker for tumor development in these malignancies (Supplementary Figure 3).

The diagnostic efficacy of KIAA1429 expression for each cancer can be assessed using the ROC curve. We identified 12 tumors with an AUC > 0.8, including ACC (0.897), BLCA (0.830), BRCA (0.891), DLBC (0.975), glioblastoma multiforme (GBM, 0.854), KIRC (0.891), LAML (0.943), LGG (0.867), LIHC (0.959), pancreatic adenocarcinoma (PAAD, 0.973), thymoma (THYM, 0.992), and UCS (0.896) (Supplementary Figure 4).

### Correlation of KIAA1429 expression with TMB, MSI

**Figure 3:**
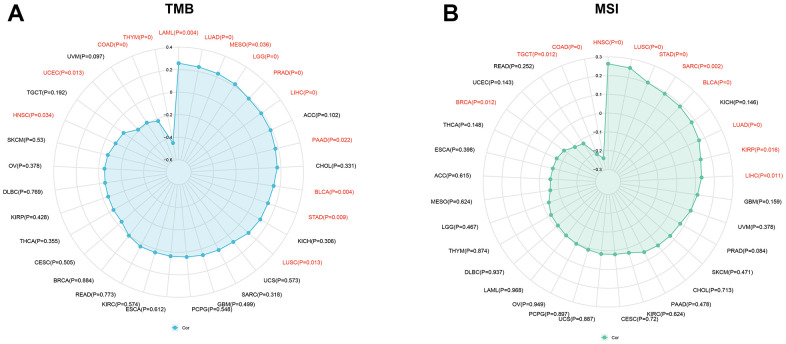
**Associations between KIAA1429 expression and tumor mutational burden (TMB), microsatellite instability (MSI).** (**A**) Correlations between KIAA1429 expression and TMB in pan-cancer. (**B**) Correlations between KIAA1429 expression and MSI in pan-cancer.

Numerous studies have demonstrated that TMB and MSI are strongly associated with immunotherapy response [20, 21]. This study aims to investigate the correlation between KIAA1429 expression and TMB as well as MSI in 33 different types of cancer. The results depicted in Figure 3A indicate a positive correlation between TMB and KIAA1429 expression in 10 cancer types. Conversely, in HNSC, LAML, uterine corpus endometrial carcinoma (UCEC), and COAD, TMB exhibits a negative correlation with KIAA1429 expression. Moreover, MSI demonstrates a positive association with KIAA1429 expression in 8 cancer types, including LUAD, LUSC, LIHC, HNSC, and STAD. However, in BRCA, COAD, and TGCT, MSI exhibits a negative connection with KIAA1429 expression (Figure 3B).

### Relationship between KIAA1429 expression and the TME

The TME comprises tumor, stromal, and immune cells, and it plays a crucial role in cell proliferation, drug resistance, metastasis, and angiogenesis [29, 30]. To evaluate the relationship between KIAA1429 expression and the components of the TME, we utilized the ESTIMATE algorithm. Supplementary Figure 5 displays the five tumors exhibiting the strongest association between KIAA1429 expression and the TME. The connection of KIAA1429 expression with TME across cancers is depicted in Supplementary Figures 6–8.

### Correlation of KIAA1429 expression with TIICs

The CIBERSORT analysis revealed a significant negative correlation between KIAA1429 and TIICs in the majority of malignancies. Six tumors, namely HNSC, LIHC, LUAD, LUSC, THCA, and THYM, exhibited the strongest correlation between KIAA1429 expression and TIICs, warranting further investigation (Supplementary Table 1). In these six tumors, KIAA1429 expression showed a positive association with memory B cells, M0 macrophages, and T follicular helper cells, while demonstrating a negative association with infiltrating naive B cells, monocytes, neutrophils, plasma cells, and resting memory CD4 T cells, except in THYM. Supplementary Figure 9 illustrates the cancers exhibiting the strongest correlation between KIAA1429 expression and TIICs. Data for other malignancies is presented in Supplementary Table 2.

**Figure 4:**
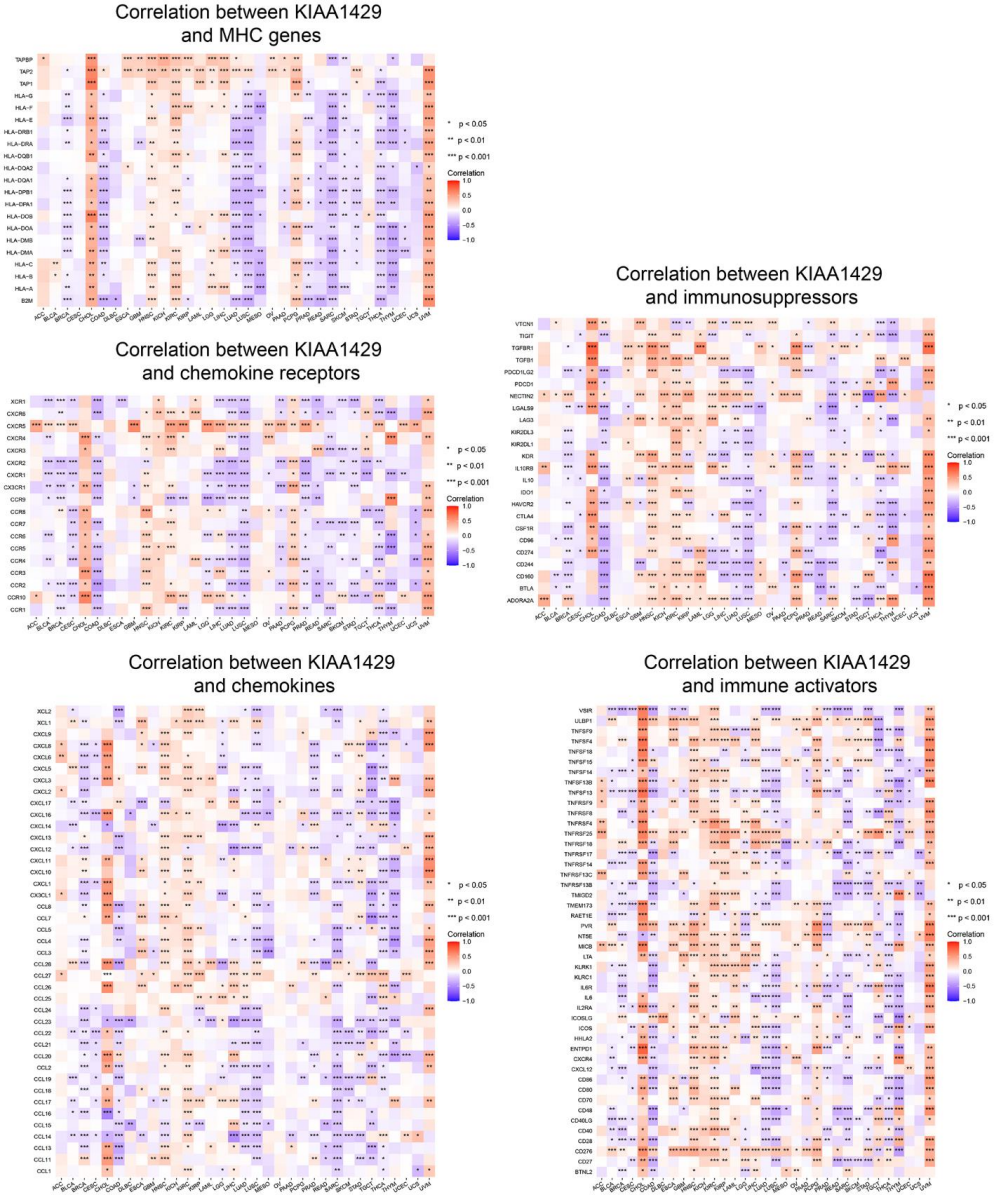
**Co-expression of KIAA1429 and immune-related genes.** *P < 0.05, **P < 0.01, ***P < 0.001.

To investigate the association between KIAA1429 expression and immune-related genes, we conducted a co-expression analysis across cancers. Figure 4 illustrates the association of KIAA1429 with a majority of immune-related genes in cholangiocarcinoma (CHOL), HNSC, KIRC, PCPG, and UVM. However, in COAD, LUAD, LUSC, MESO, PRAD, READ, SARC, SKCM, STAD, THCA, and THYM, KIAA1429 exhibits a notable negative correlation with the majority of immune-related genes.

### Correlation of KIAA1429 expression with DNA methylation

We examined the relationship between the expression of KIAA1429 and DNA methylation across various types of cancer. Supplementary Figure 10 illustrates that KIAA1429 expression is significantly correlated with promoter methylation levels in 18 cancer types. We conducted a KM analysis to investigate the impact of KIAA1429 promoter methylation levels on prognosis. As shown in Supplementary Figure 11A, high KIAA1429 methylation levels are associated with shorter OS in patients with BLCA, BRCA, ESCA, HNSC, LUSC, PRAD, STAD, and UVM. Conversely, KIRC and READ patients with high KIAA1429 methylation levels have a longer OS. Analysis of DSS data revealed that KIAA1429 methylation is a potential risk factor in BLCA, BTCA, HNSC, LUSC, PRAD, STAD, and UCEC (Supplementary Figure 11B). In terms of PFI, KIAA1429 methylation may serve as a potential risk factor in BLCA, HNSC, LUSC, STAD, and UCEC (Supplementary Figure 11C). Furthermore, Supplementary Figure 11D demonstrates that low KIAA1429 methylation levels are positively associated with shorter DFI in patients with ESCA, PRAD, and STAD.

### Enrichment analysis

**Figure 5:**
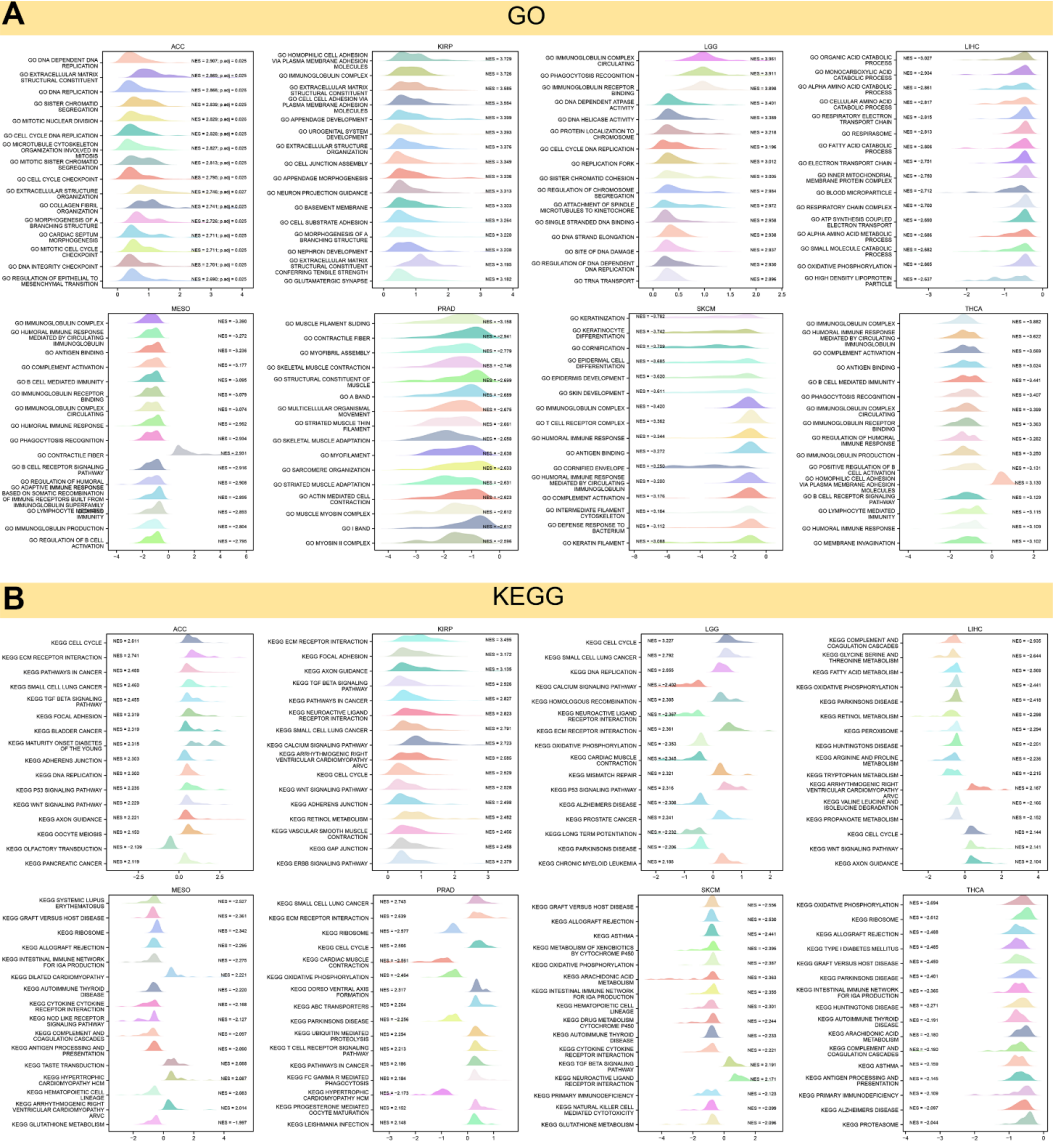
**Results of GSEA.** (**A**) GO functional annotation of KIAA1429 in pan-cancer. (**B**) KEGG pathway analysis of KIAA1429 in pan-cancer. Curves of different colors show different functions or pathways regulated in different cancers.

GSEA and GSVA were employed to investigate the biological significance of KIAA1429 expression across cancers. Figure 5A illustrates that KIAA1429 negatively regulates lymphocyte/B cell-mediated immunity, immune response, immune regulation, and B cell receptor signaling in MESO and THCA. However, KIAA1429 serves as a stimulator for cell cycle, DNA replication, and extracellular matrix remodeling in ACC, KIRP, and LGG.

The expression of KIAA1429 in MESO, SKCM, and THCA negatively regulates immune-related pathways, including autoimmune thyroid disease, primary immunodeficiency, and the intestinal immune network for IGA production. Conversely, in ACC, KIRP, LGG, LIHC, and PRAD, KIAA1429 expression positively regulates the cell cycle, ECM receptor interaction, and tumor-related pathways, including P53, TGF-β, WNT, ERBB, and calcium signaling (Figure 5B).

**Figure 6:**
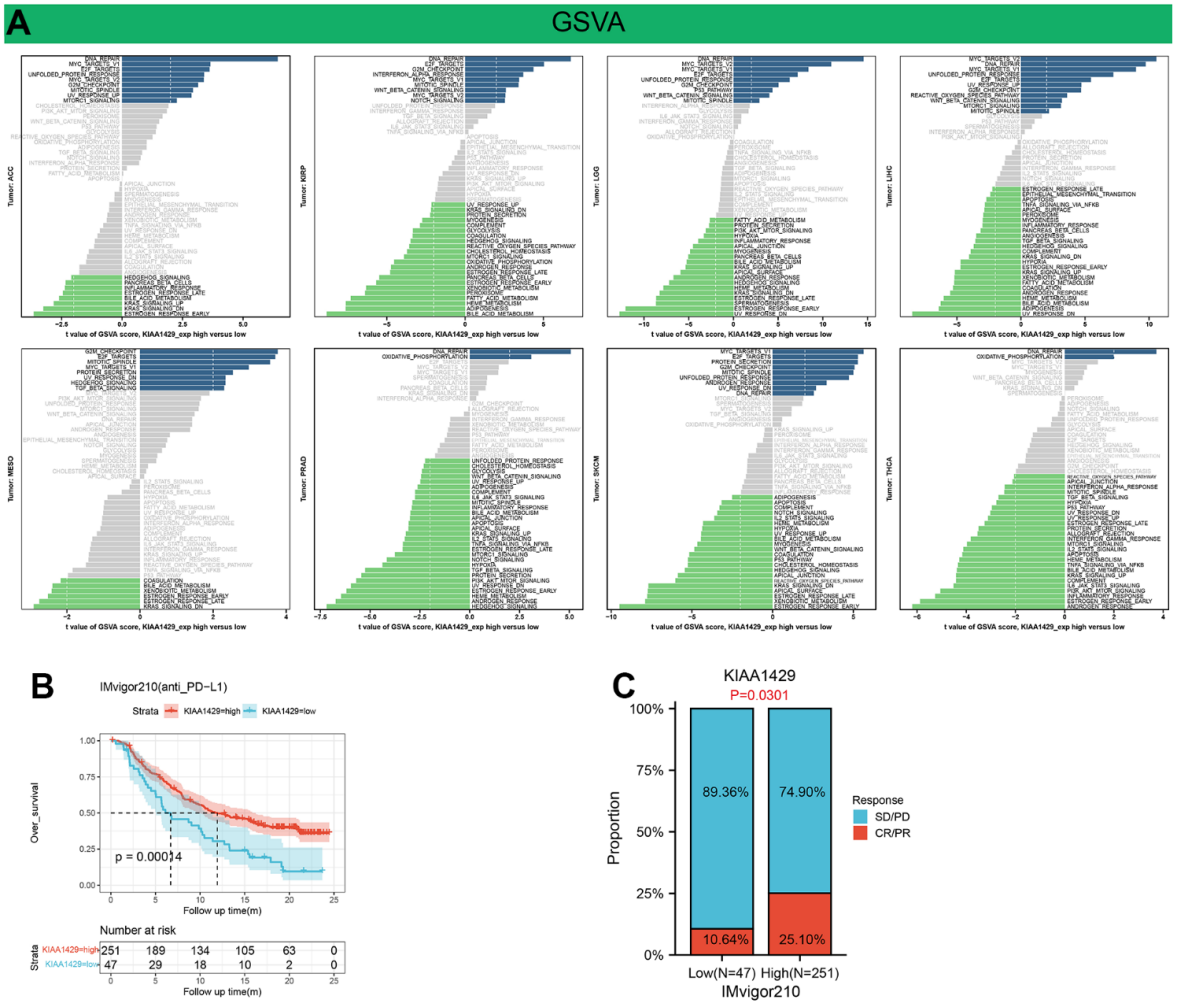
**Results of GSVA and immunotherapy prediction analysis.** (**A**) Differences in the scores for pathway activity between groups with high and low KIAA1429 expression. dn, down; UV, ultraviolet; v1, version 1; v2, version 2. (**B**) Kaplan-Meier analysis of the association between KIAA1429 expression and OS in the IMvigor210 cohort. (**C**) The proportion of BLCA patients who responded to anti-PD-L1 therapy in the low- and high-KIAA1429 subgroups.

GSVA analysis further revealed the differences in the scores for pathway activity between groups with high and low KIAA1429 expression. Figure 6A suggests that KIAA1429 is predicted to be a positive regulator for the WNT-β catenin pathway, P53 signaling, hedgehog signaling, TGF-β signaling, and cell growth-related pathways in ACC, KIRP, LGG, LIHC, MESO, SKCM, while in PRAD and THCA, KIAA1429 could be a negative regulator for TGF-β, IL2-STAT5, TNF-α, PI3K-AKT-mTOR, IL6-JAK-STAT3, and KRAS signaling.

### Immunotherapy prediction and drug sensitivity analysis

**Figure 7:**
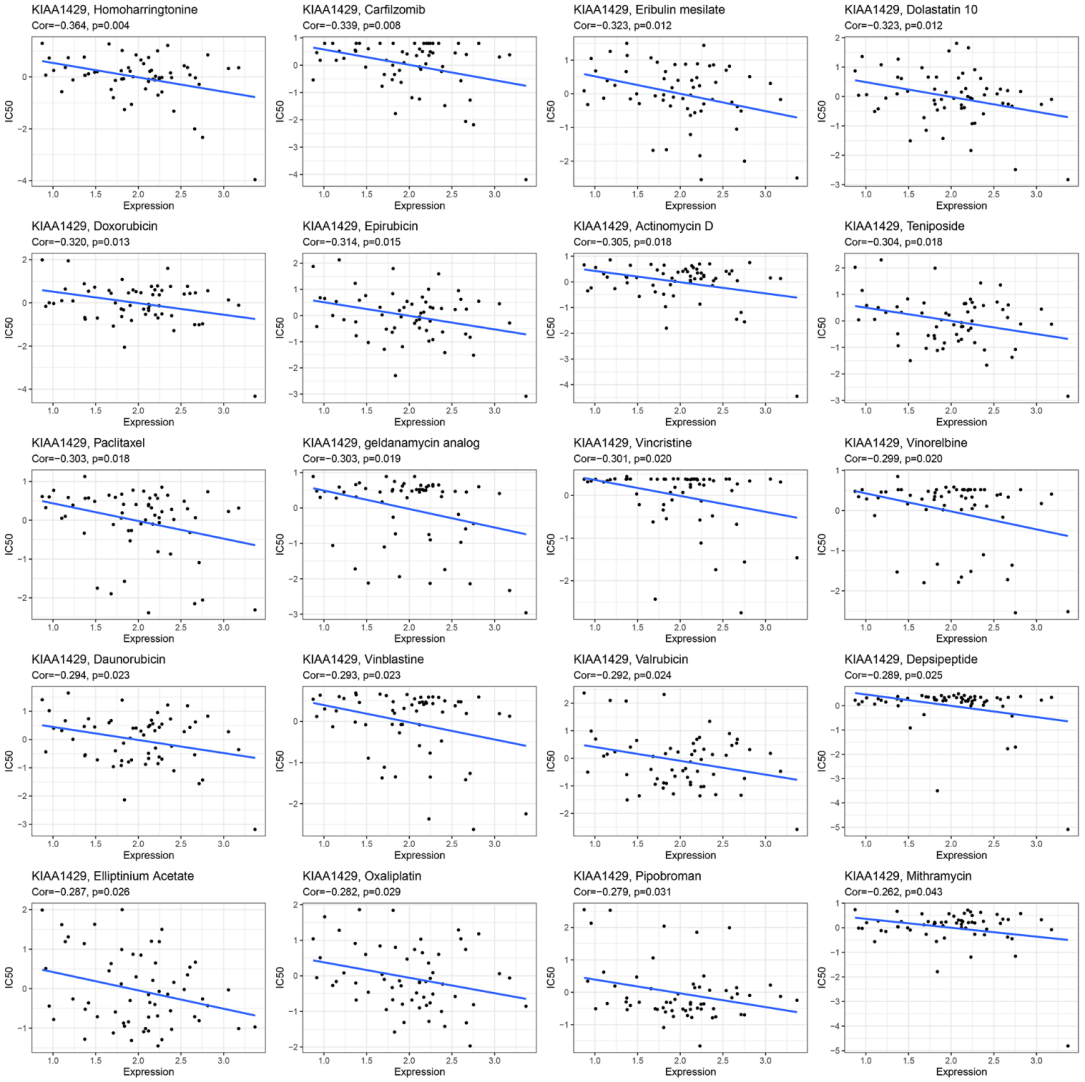
The relationship between KIAA1429 expression and expected medication response.

This study investigates the role of KIAA1429 expression in predicting the response to immunotherapy in tumor patients treated with ICIs. KM analysis indicates that elevated KIAA1429 expression is associated with shorter OS in patients with BLCA (p=1.4e-4) (Figure 6B). Figure 6C demonstrates that the group with high KIAA1429 expression has a response rate of 25.10%, whereas the group with low KIAA1429 expression exhibits a response rate of 10.64%. The significance of the difference between the high and low KIAA1429 expression groups is 0.0301. Consequently, BLCA patients with high KIAA1429 expression demonstrate increased sensitivity to immunotherapy. These findings indicate that KIAA1429 serves as a promising prognostic biomarker and predictor for immunotherapy in BLCA patients. Moreover, the analysis of drug sensitivity demonstrates a negative association between KIAA1429 expression and twenty anti-cancer drugs (Figure 7).

### RT-qPCR

**Figure 8:**
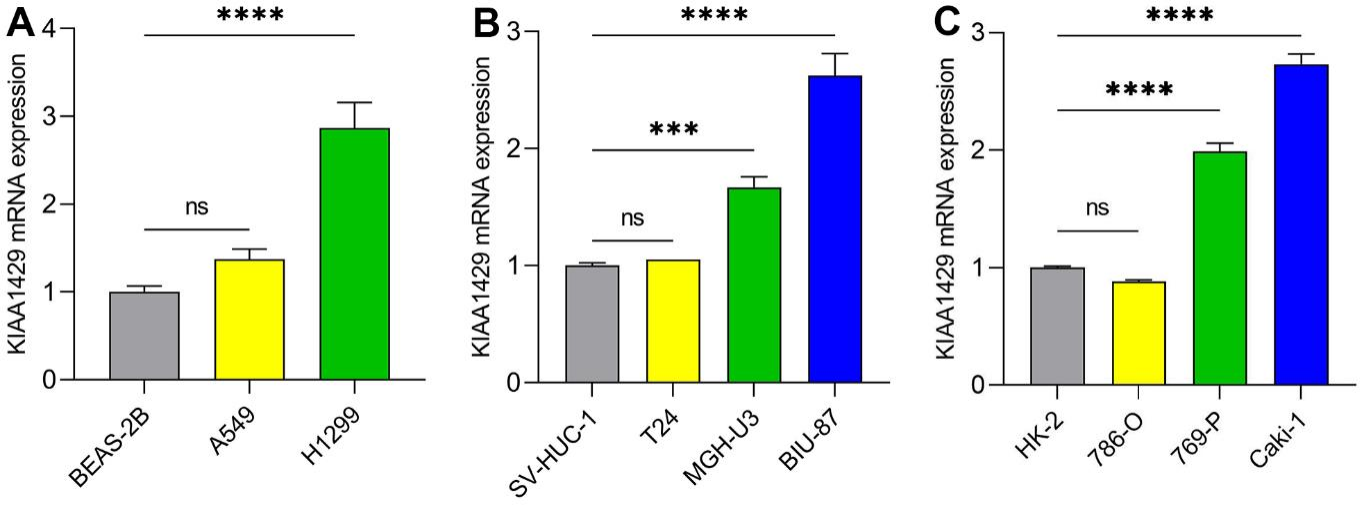
**The mRNA expression levels of KIAA1429 in different cell lines.** (**A**) LUAD cell lines, (**B**) BLCA cell lines, (**C**) KIRC cell lines. ***P < 0.001; ****P < 0.0001.

RT-qPCR was conducted to validate the expression levels of KIAA1429 mRNA in various cell lines, including LUAD cells (Figure 8A), BLCA cells (Figure 8B), and KIRC cells (Figure 8C). The data demonstrated significant overexpression of KIAA1429 in five tumor cell lines, namely H1299, MGH-U3, BIU-87, 769-P, and Caki-1. Altogether, these findings suggest that KIAA1429 holds promise as both a tumor biomarker and a therapeutic target.

## DISCUSSION

The methyltransferase KIAA1429 plays a crucial role in regulating RNA metabolism, influencing various biological processes such as tumorigenesis, obesity, and infertility through its impact on m6A methylation levels [31]. Accumulating studies suggest that KIAA1429 promotes cell growth, migration, invasion, metastasis, and drug resistance, dependent or independent of m6A modification [15, 32]. For instance, KIAA1429 promotes tumor growth and metastasis by reducing downstream gene GATA3 expression in LIHC [15]. KIAA1429 elevation negatively correlates with tumor suppressor gene DAPK3 expression in non-small cell lung cancer [33]. Mechanistically, KIAA1429 upregulates the methylation levels of DAPK3, RNA-binding protein YTHDF2/3 degrades DAPK3 mRNA by recognizing m6A sites. KIAA1429 accelerates the malignant progression of COAD by increasing the expression of SIRT1 by stabilizing its mRNA [16]. In BRCA, KIAA1429 promotes tumor progression by upregulating CDK1 expression, independent of m6A methylation [34]. Furthermore, KIAA1429 plays an oncogenic role in tumorigenesis in STAD, where mechanism analysis showed that KIAA1429 promotes c-Jun expression by stabilizing c-Jun mRNA [17].

We conducted a systematic analysis to assess the diagnostic and prognostic value of KIAA1429 across cancers, utilizing the TCGA and GTEx databases. Our findings indicate that KIAA1429 exhibits upregulation in 28 different malignancies compared to normal tissues. IHC staining of the KIAA1429 protein provided further validation of the aforementioned results. The RT-qPCR results demonstrated significant upregulation of KIAA1429 mRNA expression in several cancers, including LUAD, BLCA, and KIRC cells.

The Cox regression model indicates that KIAA1429 is a high-risk gene in LUAD and LIHC. Moreover, KM survival analysis confirms that elevated expression of KIAA1429 is associated with shorter OS in LUAD, and LIHC. In LUAD, gene amplification of KIAA1429 leads to its high expression, which is linked to a poor prognosis in patients with LUAD [18]. Furthermore, KIAA1429 is significantly upregulated in LIHC tissues and correlates with tumor volume, serum AFP levels, microvascular invasion, and TNM stage [15]. Elevated expression of KIAA1429 indicates a poorer prognosis for patients with LIHC. These findings highlight the potential of KIAA1429 as an independent prognostic biomarker in the aforementioned tumors. However, further research is needed to uncover the detailed molecular mechanisms of KIAA1429 in tumorigenesis.

Our studies revealed a strong association between KIAA1429 expression and TNM stage, as well as pathological stage, in patients with BRCA, COAD, KIRP, PRAD, UVM, KIRC, KICH, HNSC, THCA, and MESO. Particularly in COAD and BRCA, KIAA1429 exhibits significant upregulation in advanced-stage tumors compared to early-stage tumors. Therefore, KIAA1429 may serve as a potential biomarker for tumor progression in these types of cancers. The AUC suggests that the expression level of KIAA1429 has a higher diagnostic value in 12 cancer types.

TMB and MSI have emerged as promising biomarkers for immunotherapy, showing a close association with clinical outcomes [35]. Previous studies have reported that KIAA1429 expression is connected with TMB in 10 different types of cancers and with MSI in 8 different types of cancers. Consequently, KIAA1429 has the potential to serve as a predictive factor for the response rate to immunotherapy in these tumor types.

Accumulating evidence suggests that m6A methylation plays a crucial role in tumor immunity [11]. Depletion of METTL3 in macrophages enhances tumorigenesis and tumor progression by facilitating the development of an immunosuppressive microenvironment through the upregulation of infiltration levels of M1- and M2-macrophages as well as regulatory T cells (Tregs) [36]. Moreover, depletion of METTL3 in Tregs hampers Socs mRNA stability, leading to the activation of IL-2/STAT5 signaling through the inhibition of m6A methylation. This process diminishes anti-tumor immune responses within the TME [37].

The ESTIMATE analysis revealed a significant negative correlation between KIAA1429 expression and StromalScore, ImmuneScore, and ESTIMATEScore across camcers. TIICs have been shown to affect the clinical outcome of tumor patients by affecting their response to immunotherapy. Our findings demonstrate that KIAA1429 is positively associated with TIICs in 11 types of cancer and negatively associated with TIICs in 13 types of cancer. Notably, TIICs with the highest correlation to KIAA1429 expression in LIHC, COAD, were Tregs, resting memory CD4 T cells, respectively. A co-expression analysis of KIAA1429 with immune-related genes reveals that KIAA1429 expression is associated with MHC genes, chemokines, chemokine receptors, immunosuppressors, and immune activators in most types of tumors. Guo et al. found that PD-L1 was significantly overexpressed in esophageal squamous cell carcinoma (ESCC) tissue and negatively correlated with the expression level of KIAA1429 [38]. These results suggest that KIAA1429 regulates the immune microenvironment of multiple cancer types in an m6A methylation manner, which in turn affects immunotherapeutic efficacy.

Furthermore, our study revealed a positive correlation between KIAA1429 expression and DNA methylation in 18 different cancer types. Thus, the methylation level of KIAA1429 is anticipated to serve as a prognostic biomarker for specific tumor types.

GSEA and GSVA results suggested that KIAA1429 plays a pivotal role in tumor development through its involvement in essential cellular processes such as the cell cycle, DNA replication, and extracellular matrix remodeling. Moreover, KIAA1429 contributes to tumor immune suppression by influencing immune-related functions and pathways, such as lymphocyte/B cell-mediated immunity, immune response regulation, and B cell receptor signaling. The analysis of drug sensitivity demonstrated that KIAA1429 expression was negatively correlated with the IC50 values of Homoharringtonine, Carfilzomib, Eribulin mesylate, Dolastatin 10, and Doxorubicin.

In conclusion, our research demonstrates that the expression of KIAA1429 is upregulated in the majority of cancer types, and it exhibits a close correlation with the TNM stage, pathological stage, DNA methylation, and clinical outcome. KIAA1429 participates in tumor immunity by influencing TMB, MSI, and TIICs. Through enrichment analysis, we identified that KIAA1429 plays a role in tumor development by influencing key oncological pathways. However, further investigation is necessary to determine the specific role of KIAA1429 in each type of cancer. Thus, our studies indicate that KIAA1429 holds promise as a diagnostic and prognostic biomarker, with its potential to predict response to immunotherapy.

## Supplementary Material

Supplementary Figures

Supplementary Table 1

Supplementary Table 2
