# Supplementary material for: Pan-cancer analysis and experimental validation revealed the m6A methyltransferase KIAA1429 as a potential biomarker for diagnosis, prognosis, and immunotherapy
Source: Aging (Albany NY). 2023 Aug 21;15(17):8664–91. doi: 10.18632/aging.204968 (PMC10522386; doi:10.18632/aging.204968)
Supplement: Supplementary Figures [file aging-15-204968-s001.pdf]

SUPPLEMENTARY FIGURES

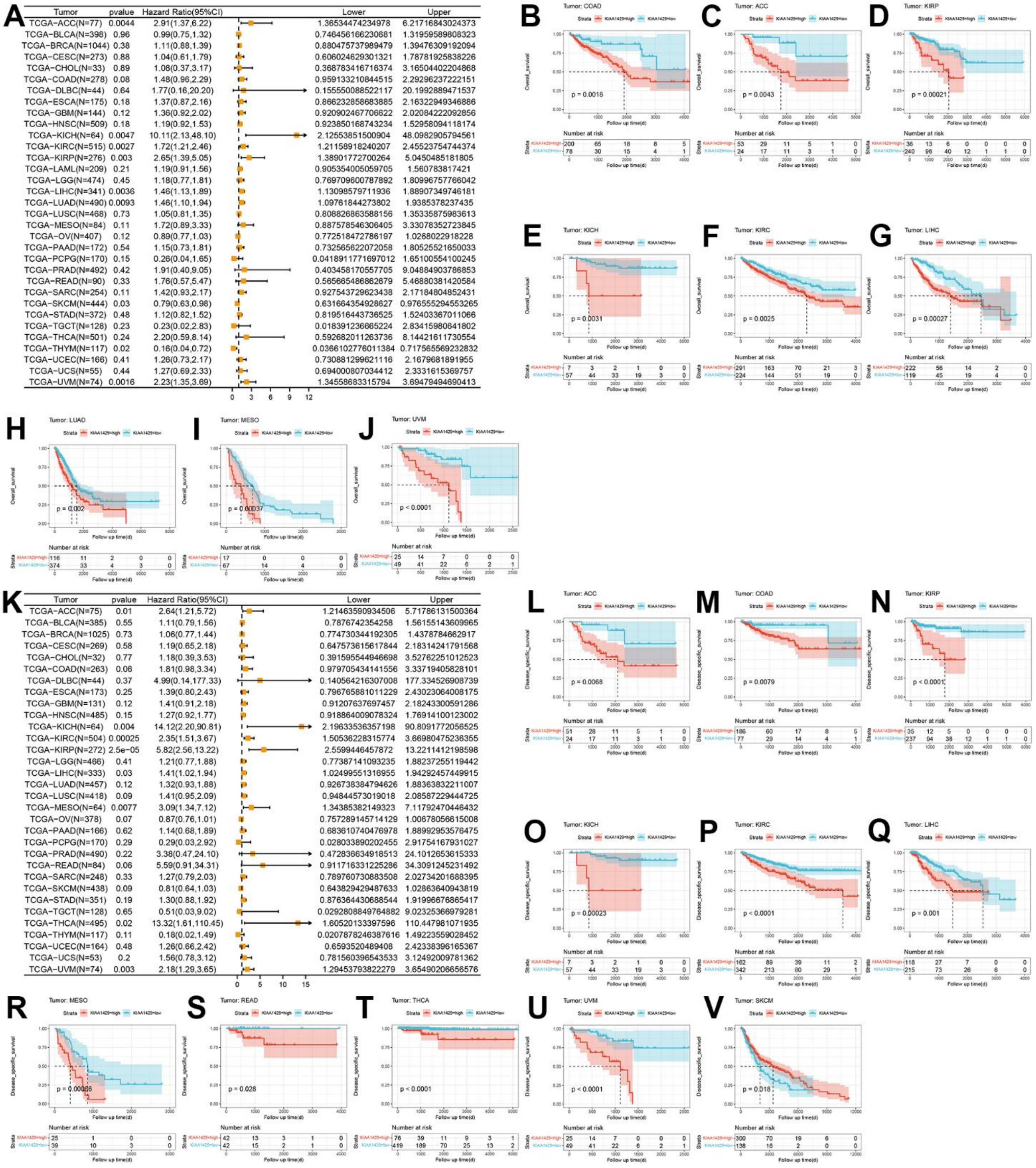

Supplementary Figure 1. Association between KIAA1429 expression levels and overall survival (OS) and disease-specific survival (DSS). (**A**) Forest plot of association of KIAA1429 expression and OS across cancers. (**B**–**J**) Kaplan-Meier analysis of the association between KIAA1429 expression and OS. (**K**) Forest plot of association of KIAA1429 expression and DSS across cancers. (**L**–**V**) Kaplan-Meier analysis of the association between KIAA1429 expression and DSS.

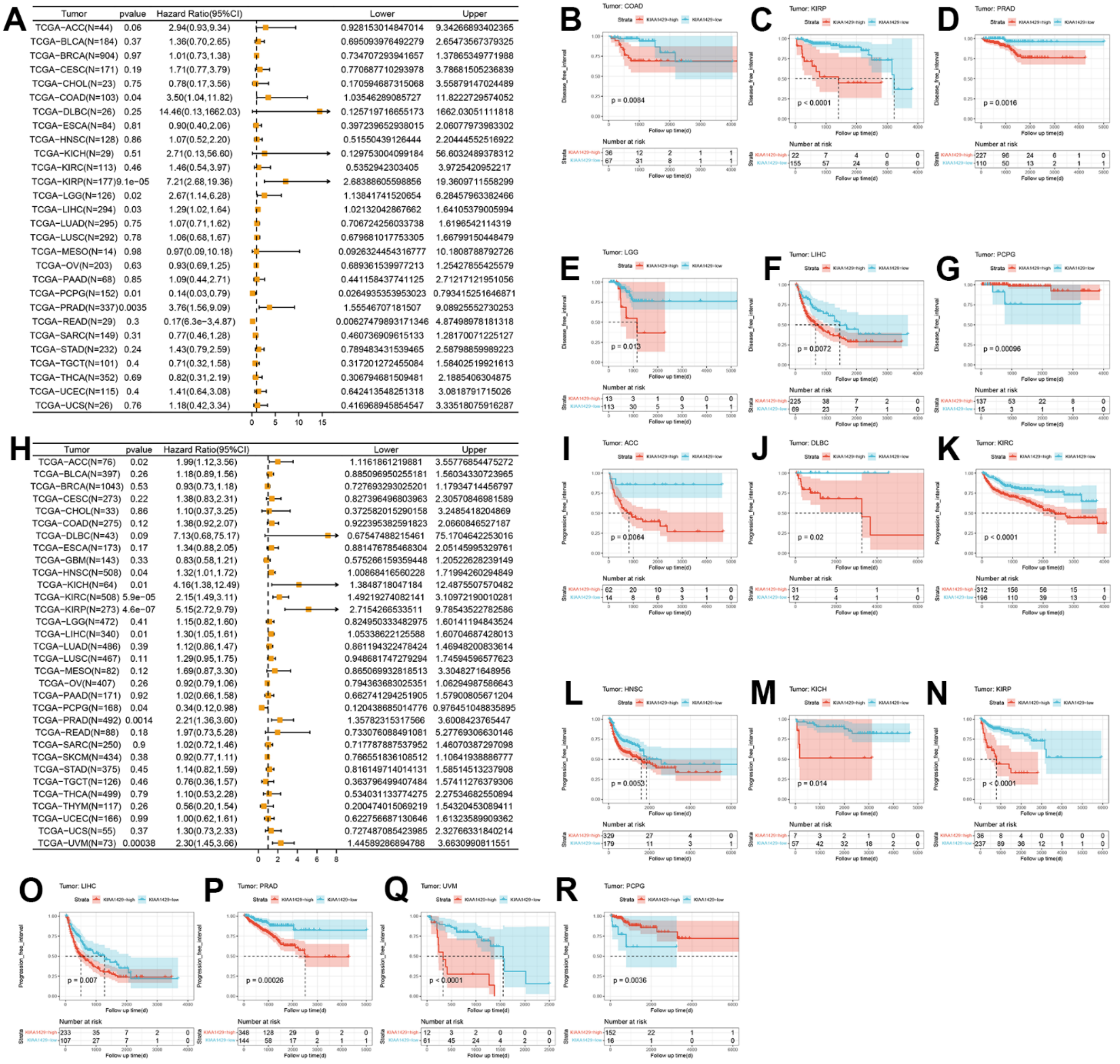

Supplementary Figure 2. Association between KIAA1429 expression levels and disease-specific survival (DFI) and progression-free interval (PFI). (**A**) Forest plot of association of KIAA1429 expression and DFI across cancers. (**B**–**G**) Kaplan-Meier analysis of the association between KIAA1429 expression and DFI. (**H**) Forest plot of association of KIAA1429 expression and PFI across cancers. (**I**–**R**) Kaplan-Meier analysis of the association between KIAA1429 expression and PFI.

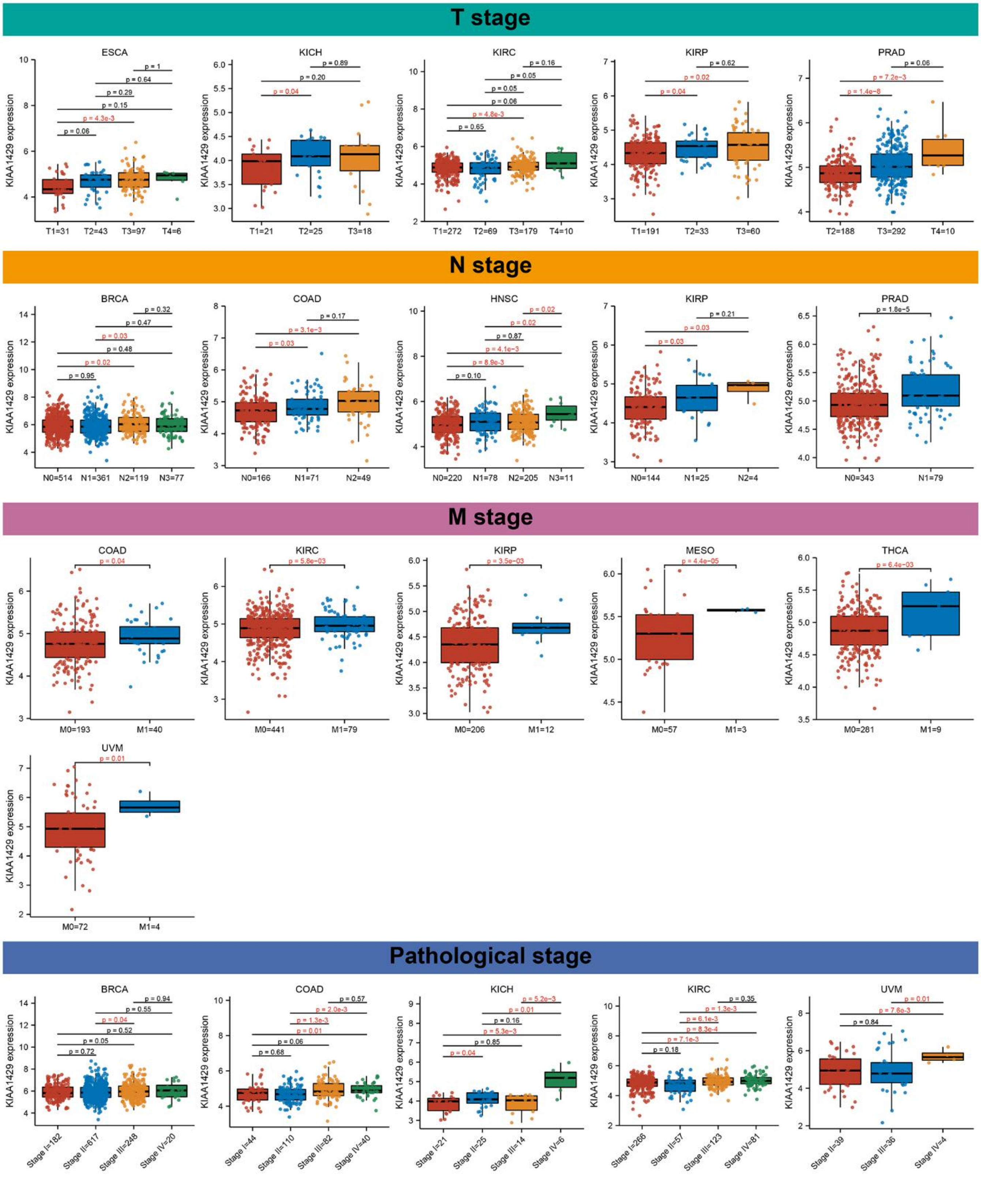

Supplementary Figure 3. Association between KIAA1429 expression and TNM stage, pathological stage.

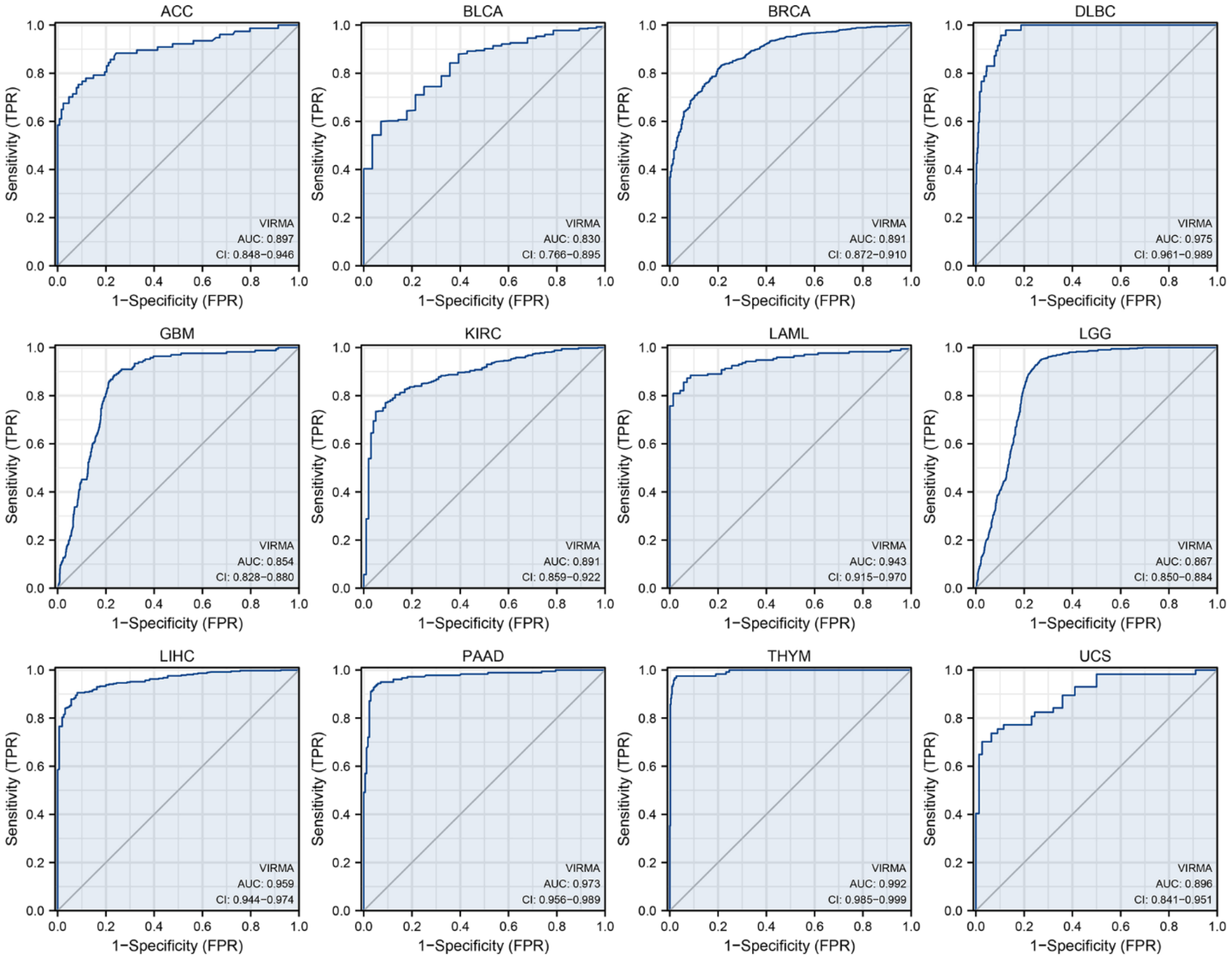

Supplementary Figure 4. ROC curves of KIAA1429 in different cancers.

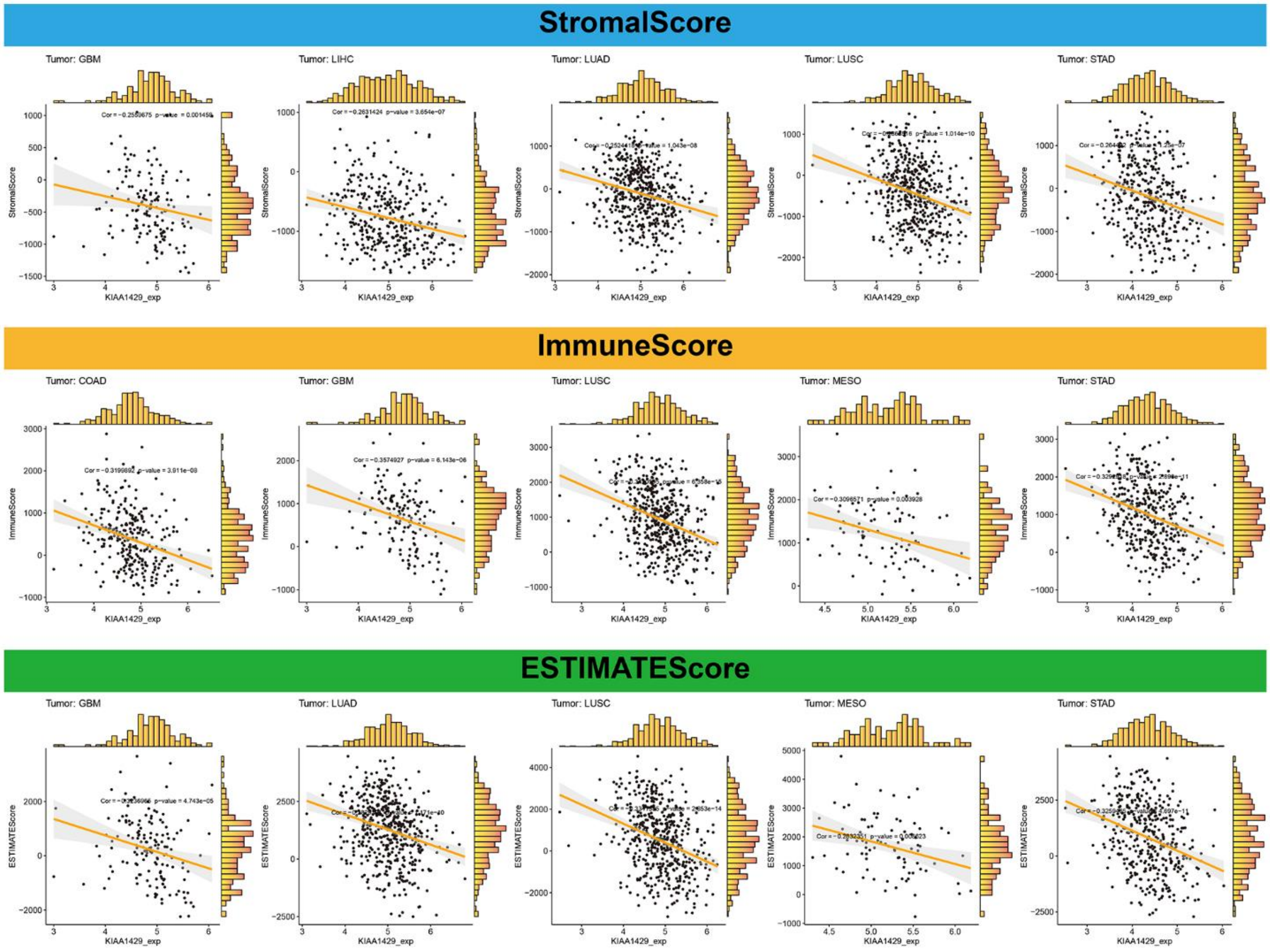

Supplementary Figure 5. Five tumors with the highest correlation coefficients between KIAA1429 expression and the tumor microenvironment.

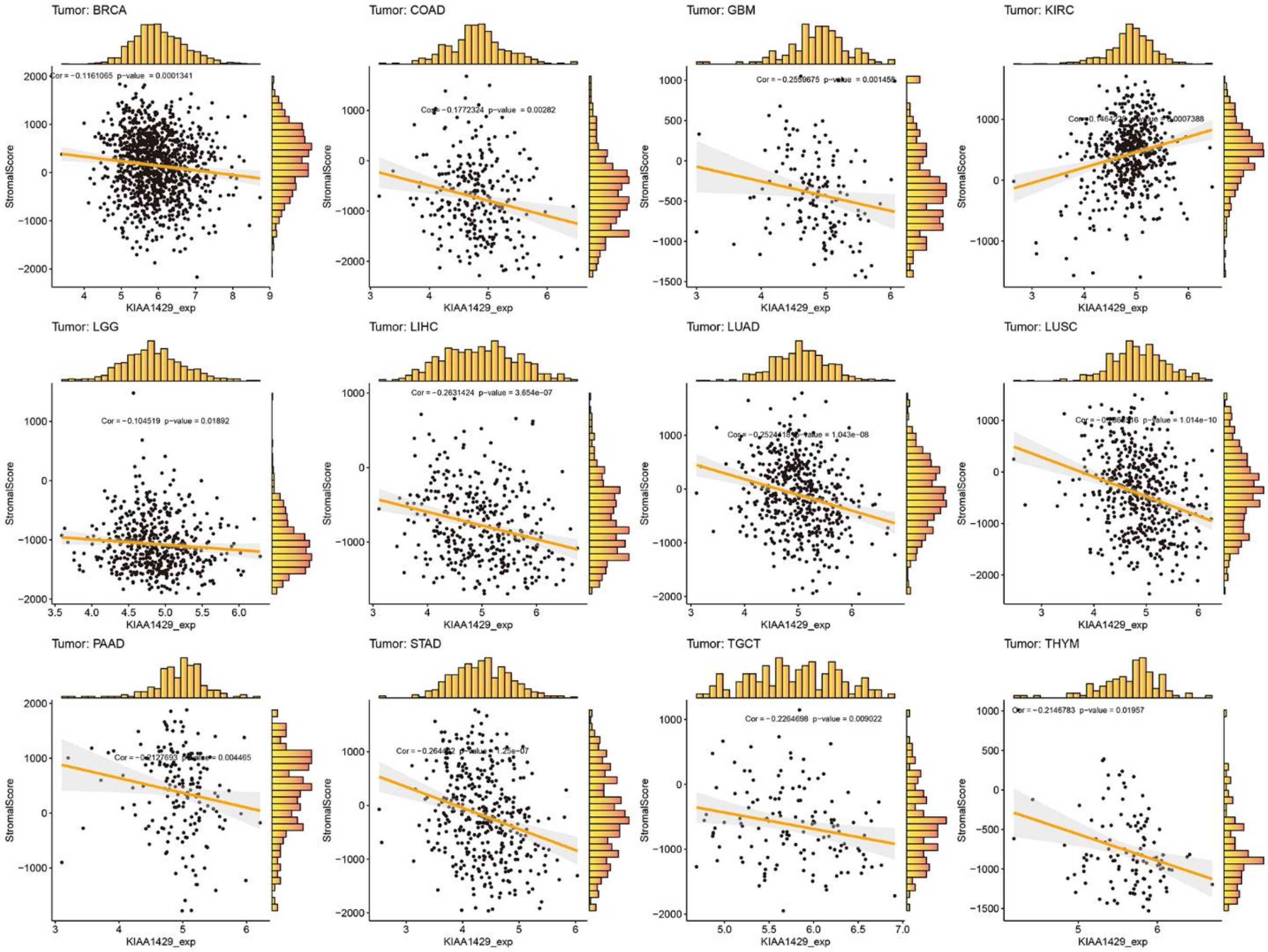

Supplementary Figure 6. Correlation of KIAA1429 expression with StromalScore in pan-cancer.

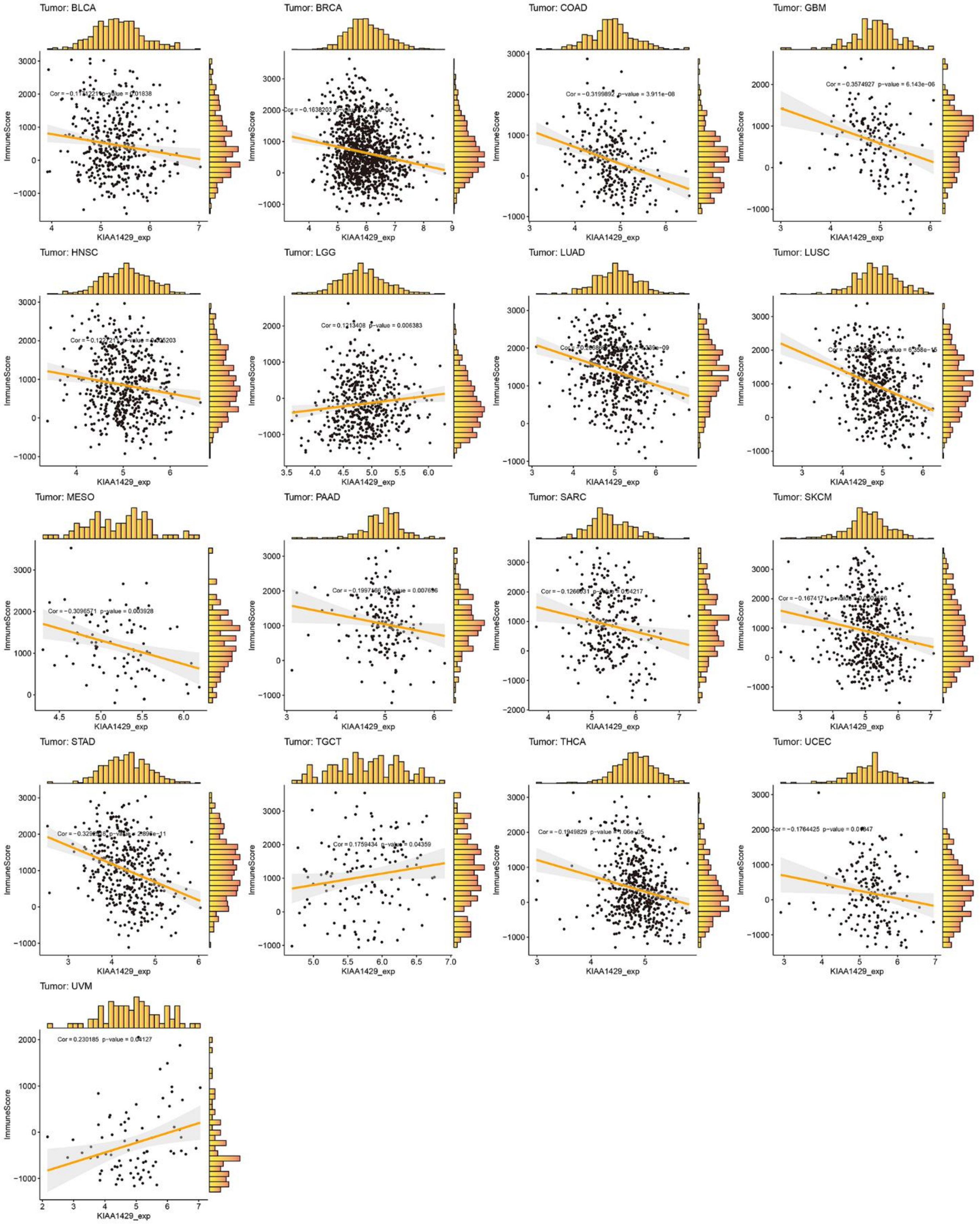

Supplementary Figure 7. Correlation of KIAA1429 expression with ImmuneScore in pan-cancer.

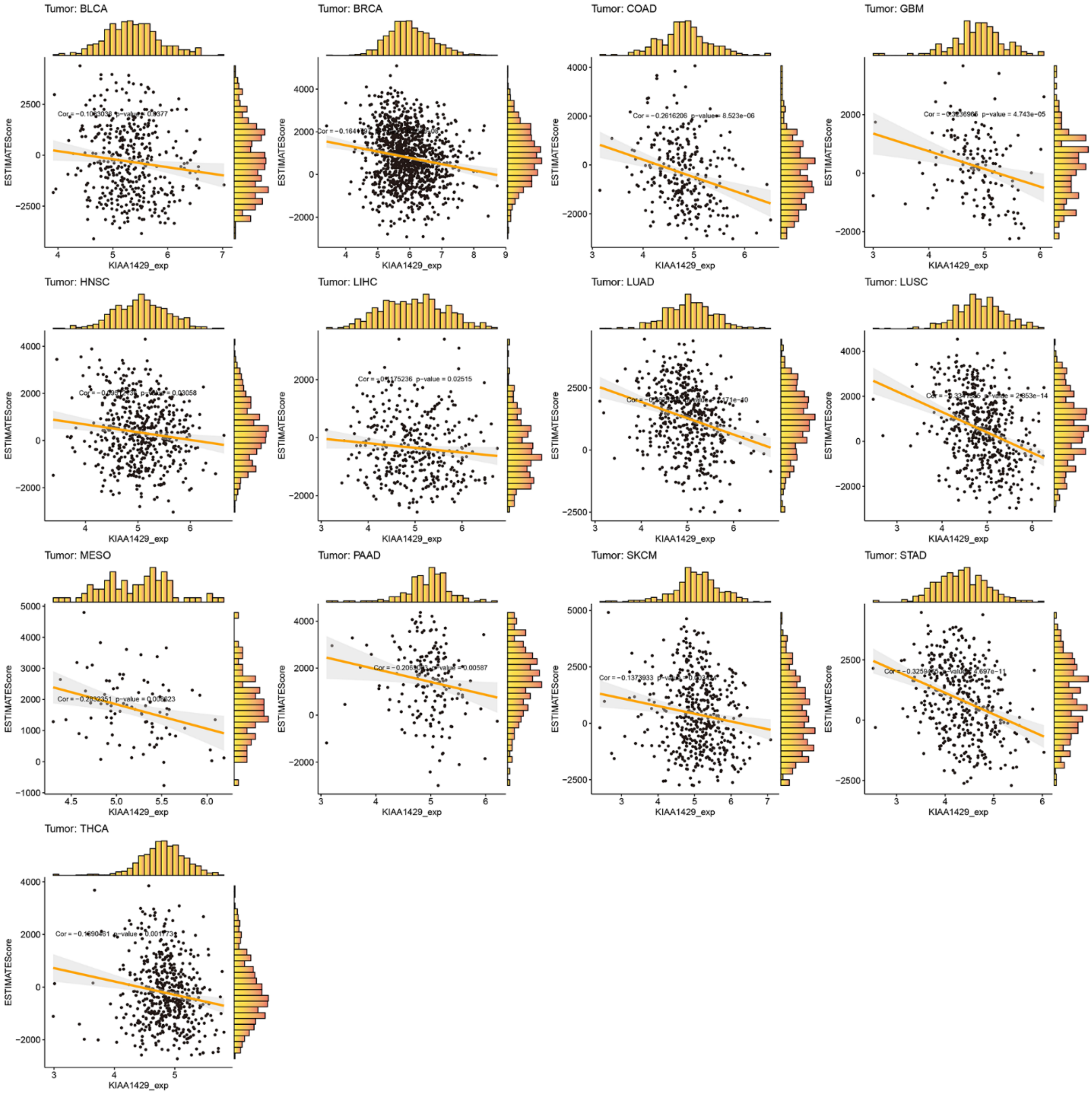

Supplementary Figure 8. Correlation of KIAA1429 expression with ESTIMATEScore in pan-cancer.

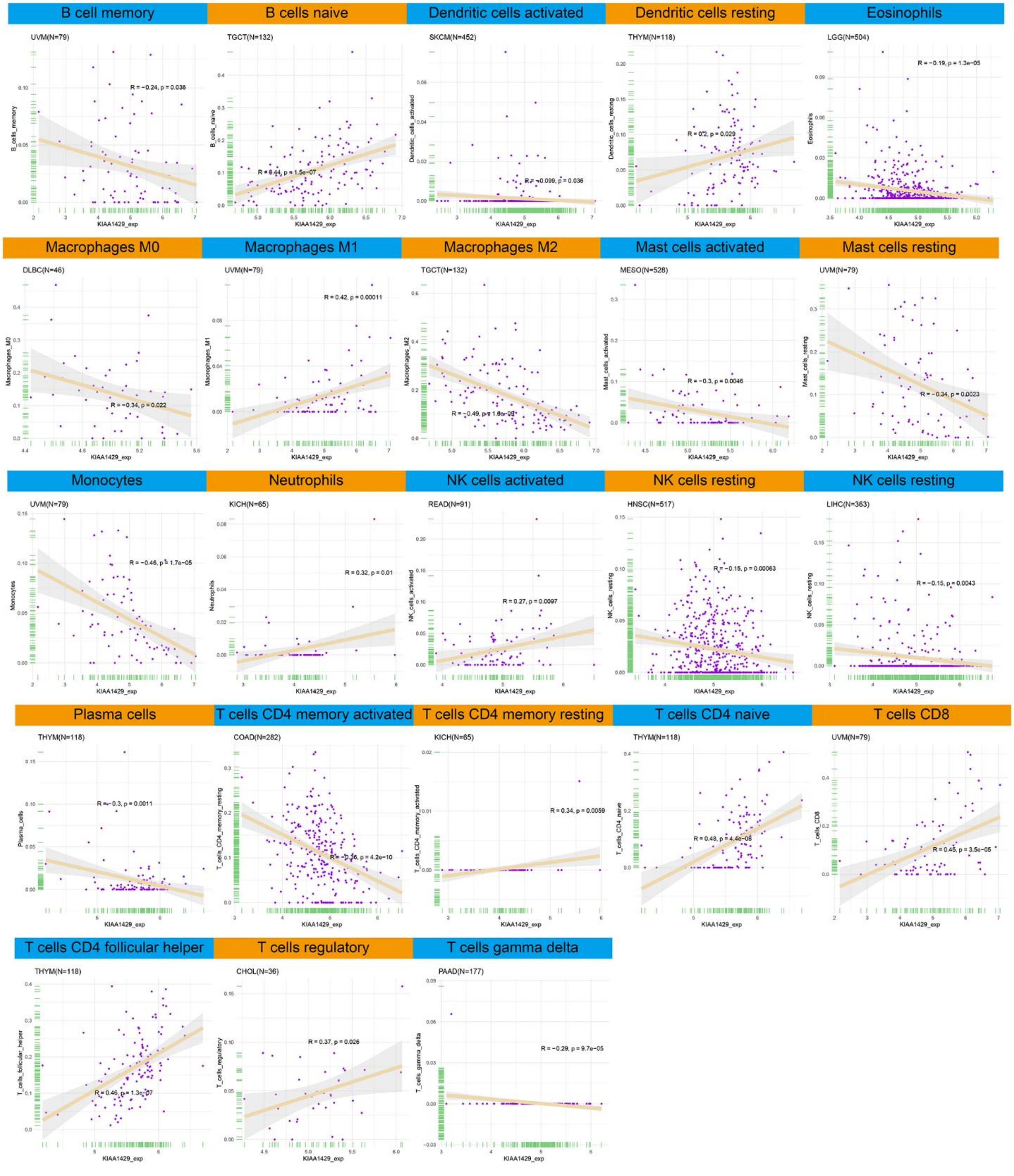

Supplementary Figure 9. Relationship between KIAA1429 expression and the infiltration scores of 22 immune cell types

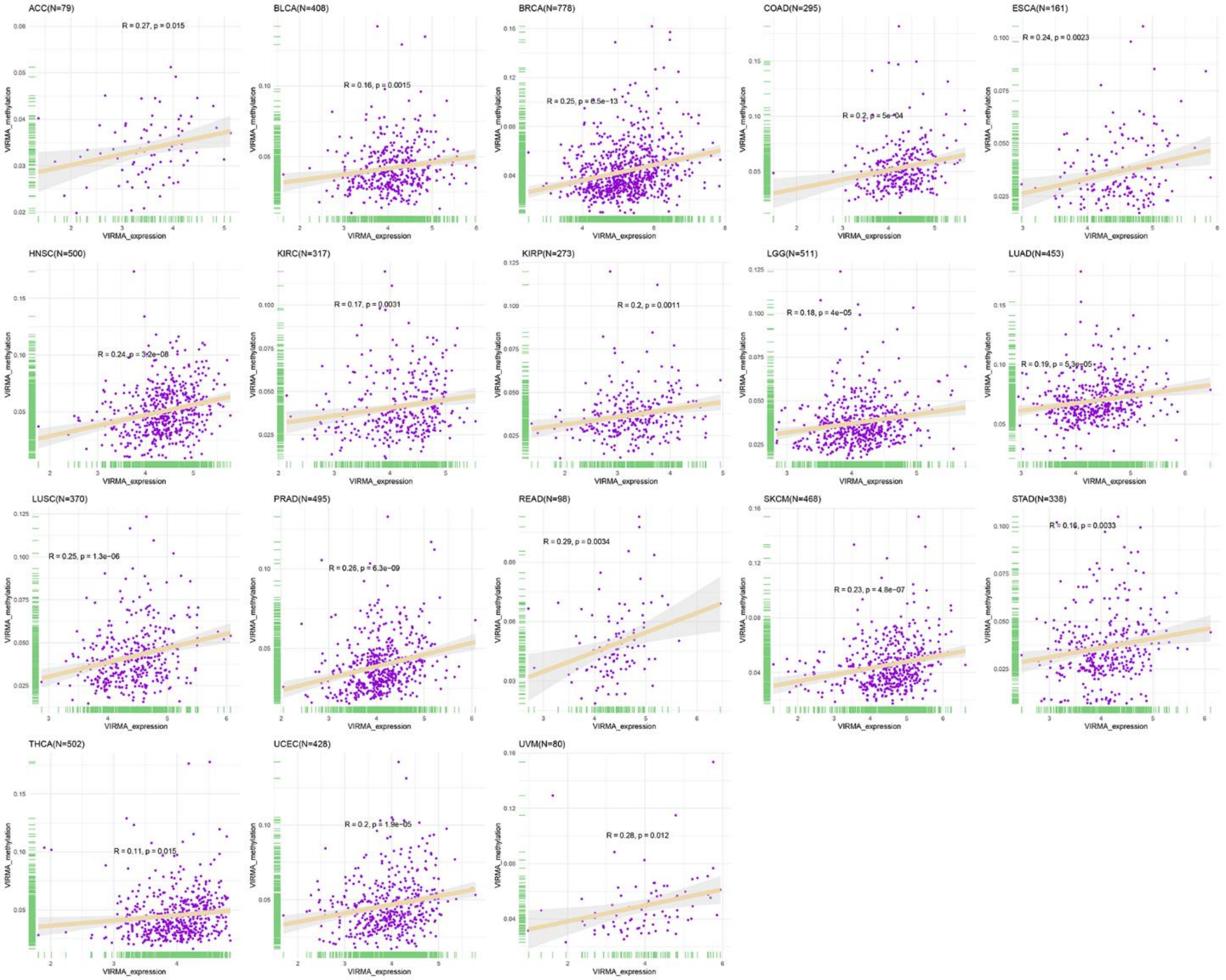

Supplementary Figure 10. Correlation between TREM2 expression and gene promoter methylation.

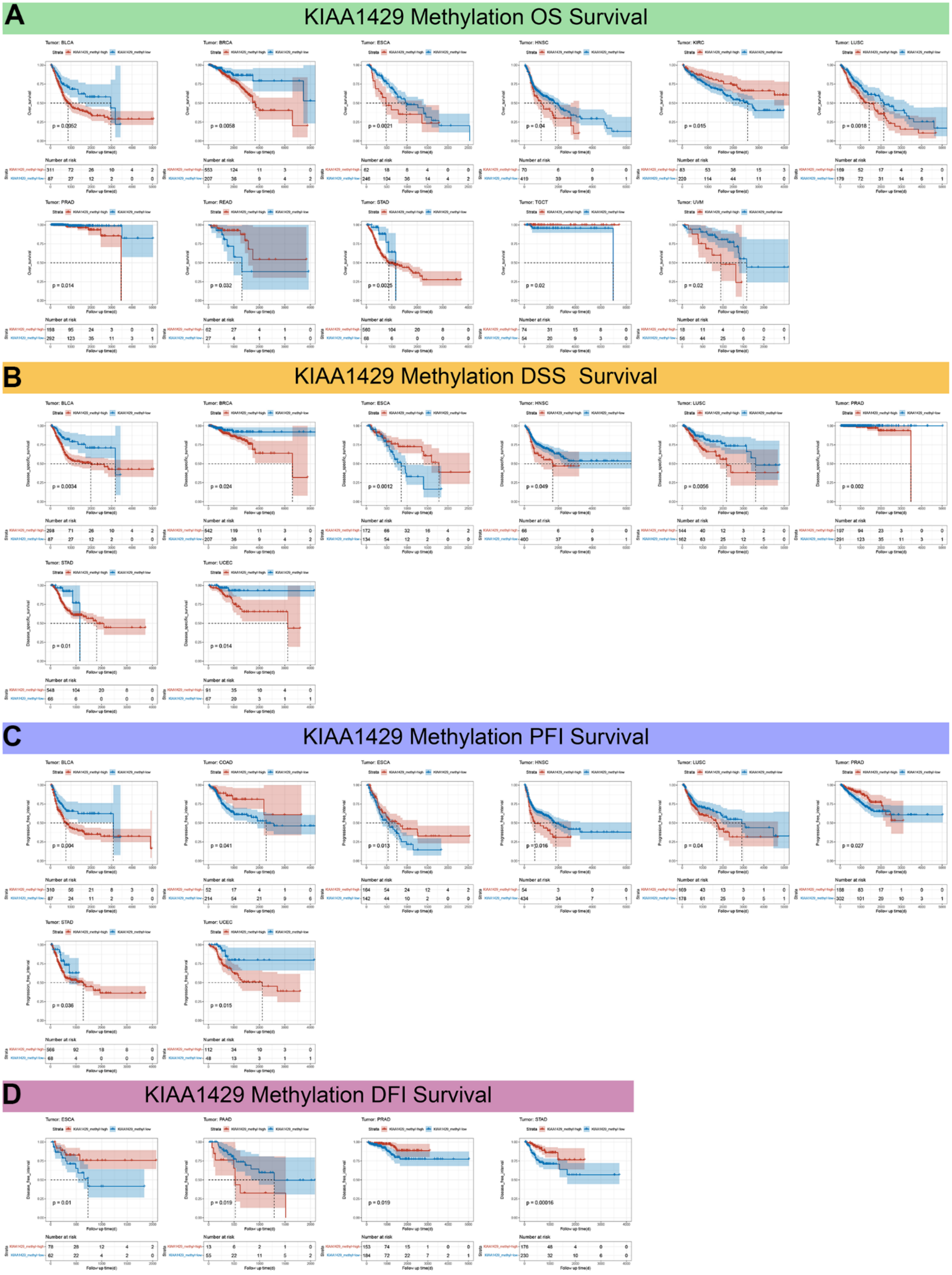

Supplementary Figure 11. Correlation between KIAA1429 promoter methylation and prognosis. (**A**) Correlation between KIAA1429 methylation and OS across cancers. (**B**) Correlation between KIAA1429 methylation and DSS across cancers. (**C**) Correlation between KIAA1429 methylation and PFI across cancers. (**D**) Correlation between KIAA1429 methylation and DFI across cancers.
