# Supplementary material for: Pan-cancer analysis and experimental validation revealed the m6A methyltransferase KIAA1429 as a potential biomarker for diagnosis, prognosis, and immunotherapy
Source: Aging (Albany NY). 2023 Aug 21;15(17):8664–91. doi: 10.18632/aging.204968 (PMC10522386; doi:10.18632/aging.204968)
Supplement: Supplementary Table 1 [file aging-15-204968-s002.pdf]

SUPPLEMENTARY TABLE

Supplementary Table 1. Relationship between KIAA1429 expression and immune cell infiltration in six cancers.

CellType	HNSC (P-value/Cor)	LIHC (P-value/Cor)	LUAD (P-value/Cor)	LUSC (P-value/Cor)	THCA (P-value/Cor)	THYM (P-value/Cor)
B cells memory	0.05	***/0.21	-0.08	0.04	**/0.11	*/-0.22
B cells naive	**/-0.14	-0.08	0.04	-0.02	**/-0.13	**/-0.27
Dendritic cells activated	-0.05	-0.06	-0.01	0.07	-0.07	0.02
Dendritic cells resting	0.08	0.06	*/-0.1	**/-0.12	0.03	*/0.2
Eosinophils	-0.04	0.04	0.03	0.02	**/0.12	-0.05
Macrophages M0	*/0.1	***/0.28	**/0.13	***/0.25	0.07	**/-0.28
Macrophages M1	0.08	-0.1	*/0.09	-0.08	**/-0.13	***/-0.39
Macrophages M2	-0.01	**/-0.16	-0.07	-0.05	0.03	***/-0.39
Mast cells activated	0.08	0.02	0.02	*/0.1	*/-0.1	**/-0.29
Mast cells resting	***/-0.15	-0.05	***/-0.21	-0.03	**/0.13	**/-0.25
Monocytes	**/-0.12	***/-0.2	*/-0.09	-0.05	0.01	-0.05
Neutrophils	***/-0.15	-0.08	*/-0.09	*/-0.11	*/-0.11	0.15
NK cells activated	**/0.13	0.08	*/0.11	***/0.15	0.06	*/-0.22
NK cells resting	***/-0.15	**/-0.15	0	*/-0.09	**/0.13	0.05
Plasma cells	*/-0.09	0.04	0.03	*/-0.09	***/-0.17	**/-0.3
T cells CD4 memory activated	0	-0.02	0.04	***/-0.18	-0.07	***/-0.4
T cells CD4 memory resting	-0.06	**/-0.14	***/-0.2	**/-0.14	*/-0.1	***/-0.32
T cells CD4 naive	0.09	0	NA	*/0.1	0.06	***/0.48
T cells CD8	-0.04	-0.01	*/0.1	*/-0.1	-0.06	0.09
T cells follicular helper	0.07	**/0.15	***/0.19	***/0.17	0.07	***/0.46
T cells gamma delta	0	0.06	-0.01	0	*/0.09	NA
T cells regulatory (Tregs)	-0.07	***/0.31	0.07	0.01	-0.03	0.17

*$P < 0.05$, **$P < 0.01$, and ***$P < 0.001$.
